# 194. Real-World Impact of the 2021 IDSA/SHEA CDI Guidelines: Shifts in Treatment, Outcomes, and Healthcare Costs

**DOI:** 10.1093/ofid/ofaf695.069

**Published:** 2026-01-11

**Authors:** Angela Wu, Shreya Jain, Mujeeb Basit, Mayar Al Mohajer

**Affiliations:** Baylor College of Medicine, Houston, TX; Baylor College of Medicine, Houston, TX; UT Southwestern Medical Center, Dallas, Texas; Baylor College of Medicine, Houston, TX

## Abstract

**Background:**

*Clostridioides difficile* infection (CDI) accounts for 450,000 cases and $6.3 billion in healthcare costs annually in the US. The June 2021 IDSA/SHEA guideline update recommended fidaxomicin over vancomycin for initial and recurrent CDI and adjuvant bezlotoxumab for high-risk patients. We evaluated the impact of this update on CDI management, outcomes, and healthcare utilization.

**Figure:**
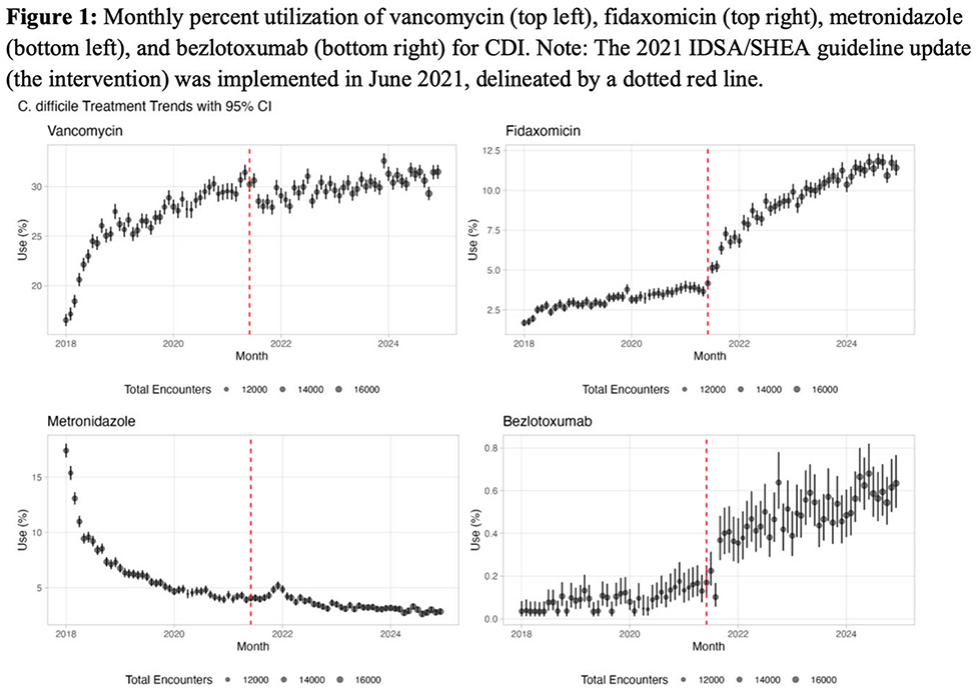

**Figure:**
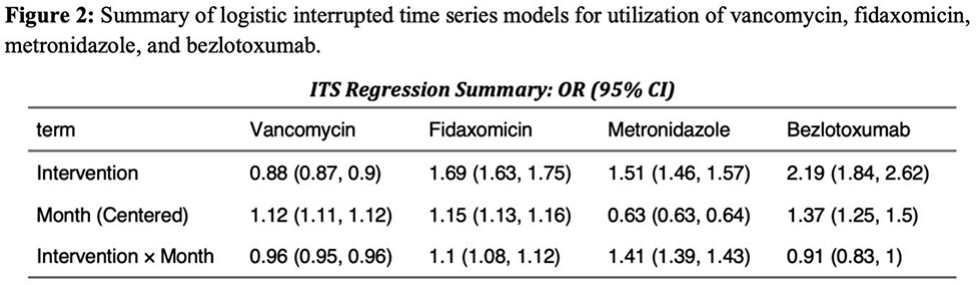

**Methods:**

We conducted a retrospective study using Epic Cosmos (Epic EHR data, US only, 2018-2024), including all adult inpatient/outpatient encounters with a CDI diagnosis. An interrupted time series analysis evaluated trends in monthly utilization of enteral fidaxomicin, vancomycin, metronidazole, and IV bezlotoxumab; 30-day CDI recurrence (logistic regression); mean length of stay (LOS) (linear regression); and total monthly CDI-related costs (linear regression; including antibiotics, LOS, and recurrence-related readmissions) before (Jun 2018-May 2021) and after (July 2021-Dec 2024) the guideline update.

**Figure:**
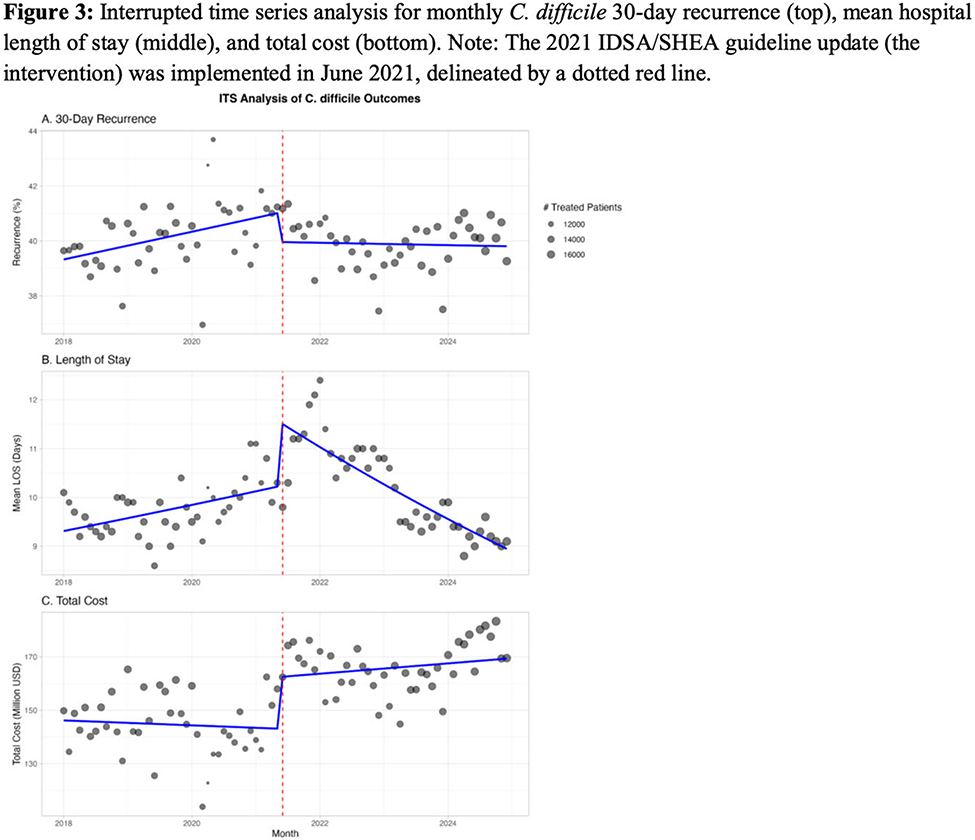

**Figure:**
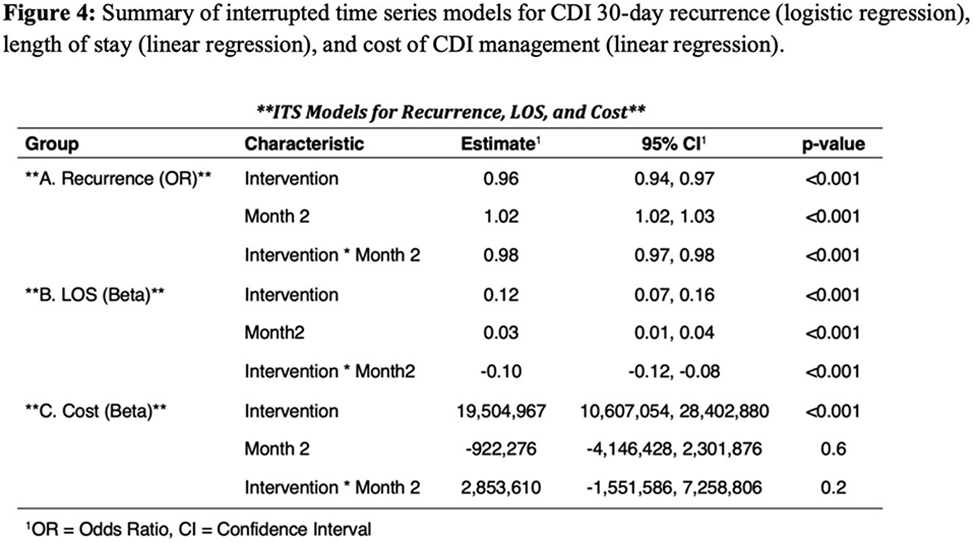

**Results:**

We identified 1,204,726 CDI encounters. Following the guideline update, fidaxomicin, vancomycin, and bezlotoxumab use increased (3.1% to 9.6%; 26.4% to 30.1%, and 0.1% to 0.5%, respectively), while metronidazole use decreased (6.6% to 3.5%, Figures 1-2). The guideline update was associated with an immediate 4% reduction in the odds of 30-day recurrence (OR 0.96, 95% CI 0.94-0.97, p< 0.001) followed by a flattening in recurrence trend post-intervention (interaction OR 0.98, p< 0.001, Figures 3-4). Mean LOS increased immediately (β=0.12 log-days, 95% CI 0.07-0.16, p< 0.001), but declined significantly over time (interaction β=–0.10, p< 0.001). Total monthly cost increased sharply post-intervention (β=$19.5 million, 95% CI: $10.6M–28.4M, p< 0.001); but slopes changes were not significant (interaction β=$2.85M, p=0.2).

**Conclusion:**

Adoption of the updated CDI guidelines was associated with reduced recurrence and declining LOS. Although monthly costs increased substantially following the intervention, there was no significant ongoing upward trend. These findings highlight the clinical benefits of guideline implementation, while emphasizing the need to balance improved outcomes against higher sustained treatment costs.

**Disclosures:**

All Authors: No reported disclosures

